# Systematic review: Nurses' safety attitudes and their impact on patient outcomes in acute‐care hospitals

**DOI:** 10.1002/nop2.1063

**Published:** 2021-09-19

**Authors:** Faisal Khalaf Alanazi, Jenny Sim, Samuel Lapkin

**Affiliations:** ^1^ School of Nursing University of Wollongong Wollongong NSW Australia; ^2^ School of Nursing & Midwifery University of Newcastle Callaghan NSW Australia; ^3^ Australian Health Services Research Institute University of Wollongong Wollongong NSW Australia

**Keywords:** adverse events, nurses, nursing, patient outcomes, safety attitude, safety climate, safety culture

## Abstract

**Aims:**

The aim of this review was to synthesize the best available evidence on the impact of nurses' safety attitudes on patient outcomes in acute‐care hospitals.

**Design:**

Systematic review with a narrative synthesis of the available data.

**Data sources:**

Data sources included MEDLINE, Cumulative Index of Nursing and Allied Health Literature, Scopus and Web of Science Core Collection. Studies published up to March 2021 were included.

**Review Methods:**

This review was conducted using guidance from the Joanna Briggs Institute for Systematic Reviews and reported as per the Preferred Reporting Items for Systematic Review and Meta‐Analyses guidelines.

**Results:**

A total of 3,452 studies were identified, and nine studies met the inclusion criteria. Nurses with positive safety attitudes reported fewer patient falls, medication errors, pressure injuries, healthcare‐associated infections, mortality, physical restraints, vascular access device reactions and higher patient satisfaction. Effective teamwork led to a reduction in adverse patient outcomes. Most included studies (*N* = 6) used variants of the Hospital Survey on Patient Safety Culture to assess nurses' safety attitudes. Patient outcomes data were collected from four sources: coded medical records data, incident management systems, nurse perceptions of adverse events and patient perceptions of safety.

**Conclusion:**

A positive safety culture in nursing units and across hospitals resulted in fewer reported adverse patient outcomes. Nurse managers can improve nurses' safety attitudes by promoting a non‐punitive response to error reporting and promoting effective teamwork and good communication.

**Impact Statement**

**What does this paper contribute to the wider global clinical community?**

- Nurses with positive safety attitudes report fewer adverse events; and units and hospitals with a positive safety culture have improved patient outcomes.
- Effective teamwork and communication among nurses lead to a reduction in the occurrence of adverse patient outcomes.
- Nurse managers play an important role in promoting a positive safety culture which in turn improves nurses' safety attitudes and ultimately patient outcomes.

## 1 INTRODUCTION

All nurses and healthcare workers have a professional responsibility to improve patient outcomes and prevent adverse events. Nurses are considered a key patient safety link between patients and other health professionals and have an important role in promoting safety issues and improving patient outcomes (Sim et al., 2019). Individual nurses hold attitudes towards safety practices that may influence patient outcomes (Han et al., 2020). These attitudes are referred to as safety attitudes and relate to an individual's beliefs, perceptions, feelings or thinking towards safety practices, procedures and policies (Sexton et al., 2006). When individual nurses have positive safety attitudes, a strong safety culture develops (Ellis et al., 2020). A positive safety culture in healthcare involves reporting of errors, a non‐punitive response to error, management support and commitment to investigate error, effective communication and organizational learning (Lee et al., 2019). Conversely, fear of reporting errors is an indicator of poor safety culture as the identification and investigation of errors are considered critical in improving patient outcomes by developing strategies aimed at preventing reoccurrence of similar incidents (World Health Organization, 2021). Therefore, nurses' safety attitudes may have a significant impact on patient outcomes and patient safety.

### 1.1 Background

Hospital or unit safety culture consists of the collective safety attitudes of staff. A commonly used definition of safety culture is “the product of individual and group values, attitudes, perceptions, competencies, and patterns of behaviour that determine the commitment to, and the style and proficiency of, an organization's health and safety management” (Waterson, 2014, p. 68–69). The term “safety climate” is frequently used interchangeably with the term “safety culture” (Hogden et al., 2017). Individual safety attitudes are most frequently measured by Likert scale self‐report questionnaires (Ellis et al., 2020). A number of instruments are used to evaluate safety culture and/or safety attitudes in healthcare settings (Hogden et al., 2017). The most commonly used instruments are the Hospital Survey on Patient Safety Culture (HSOPSC) and the Safety Attitudes Questionnaire (SAQ; Ellis et al., 2020). Both the HSOPSC and the SAQ are valid and reliable and have been translated into multiple languages (Hogden et al., 2017; Okuyama et al., 2018). For the purpose of this review, the term safety attitudes was used to describe an individual nurse's safety attitudes and aggregated scores at the unit or hospital level will be referred to as safety culture.

Patient outcomes can be defined as the changes in, or maintenance of, the patient's health‐related state and include measures such as adverse events and patient satisfaction with care (Liu et al., 2014). Patient outcomes data can be obtained from administrative datasets, which include incident management systems and discharge datasets, and from staff and/or patient surveys (Al‐ghraiybah et al., 2021; Sim et al., 2018). Data obtained from administrative datasets are considered the “gold standard” because they use routinely collected data and can include large sample sizes (Sim et al., 2019). However, nurse perceptions of the frequency of adverse events are also widely used as nurses are reliable providers of information about patient outcomes and are aware of patient safety issues (Lake et al., 2016; Lee & Scott, 2018).

Previous research has identified that nurse staffing and the nursing work environment can impact on patient outcomes (Lee et al., 2018; Stalpers et al., 2015). Nurses' safety attitudes are also thought to influence patient outcomes (Han et al., 2020). The RN4CAST research programme in Europe (conducted in 243 hospitals in six countries) identified that one in three nurses (*N* = 13,077 nurses) were reported to have poor safety attitudes (Aiken et al., 2017). Other studies have identified that nurses' poor safety attitudes are associated with negative patient outcomes (Han et al., 2020; Lee et al., 2018). Given that nurses comprise the largest percentage of the hospital workforce (Sim et al., 2019), the impact of nurses' safety attitudes on patient outcomes requires further study.

## 2 THE REVIEW

### 2.1 Aim

The aim of this review was to synthesize the best available evidence on the impact of nurses' safety attitudes on patient outcomes in acute‐care hospital settings. The question guiding this review was: “what is the impact of nurses' safety attitudes on patient outcomes in acute care hospitals?”

### 2.2 Design

This systematic review was conducted in accordance with guidance from the Joanna Briggs Institute (JBI) for Systematic Reviews (Aromataris & Munn, 2020) and reported as per the Preferred Reporting Items for Systematic Review and Meta‐Analyses (PRISMA) guidelines (Page et al., 2021). The PICO elements (Population; Intervention; Comparator; Outcome) were used to formulate the review question (see Table 1) (Aromataris & Munn, 2020). A search of the PROSPERO database identified that no systematic reviews on this topic had been previously conducted or were currently in progress. The review protocol was registered a priori with PROSPERO in July 2020 (CRD42020159074).

**TABLE 1:** PICO framework

PICO	
Population	Nurses in acute‐care hospitals
Intervention	Safety attitudes as measured by validated instruments such as Hospital Survey on Patient Safety Culture (HSOPSC) and Safety Attitudes Questionnaire (SAQ)
Comparator	Not applicable
Outcome	Patient outcomes such as medication errors, pressure injuries, and falls

### 2.3 Search method

The search strategy consisted of two phases. The first involved searches in MEDLINE (EBSCO Host), Cumulative Index of Nursing and Allied Health Literature (CINAHL, EBSCO Host), Scopus and Web of Science Core Collection. The second phase examined the reference lists of all included studies to identify any additional studies relevant to the review. The following search terms were used in all databases: “safety attitude*” OR “safety culture” OR “safety climate” AND “nurs*” AND “patient outcome*”. The search strategy for CINAHL has been documented in PROSPERO. The initial search was conducted with the assistance of a health librarian in 2020 and then updated in March 2021.

### 2.4 Inclusion and exclusion criteria

Studies were included if they were published in English, collected data from nurses working in acute‐care hospitals and explored the impact of nurses' safety attitudes on patient outcomes. Studies that examined nurses' safety attitudes as part of the interdisciplinary team were included if data from nurses could be extracted. Studies examining safety attitudes, safety culture or safety climate were included. In this review, acute‐care hospitals were defined as general, non‐specialist hospitals with surgical and medical facilities that provide active diagnosis, care and treatment of a broad range of acute conditions including trauma and injuries (Hirshon et al., 2013). No restrictions were applied to qualifications, education or experience of nurses in the included studies.

### 2.5 Search outcomes

**FIGURE 1:**
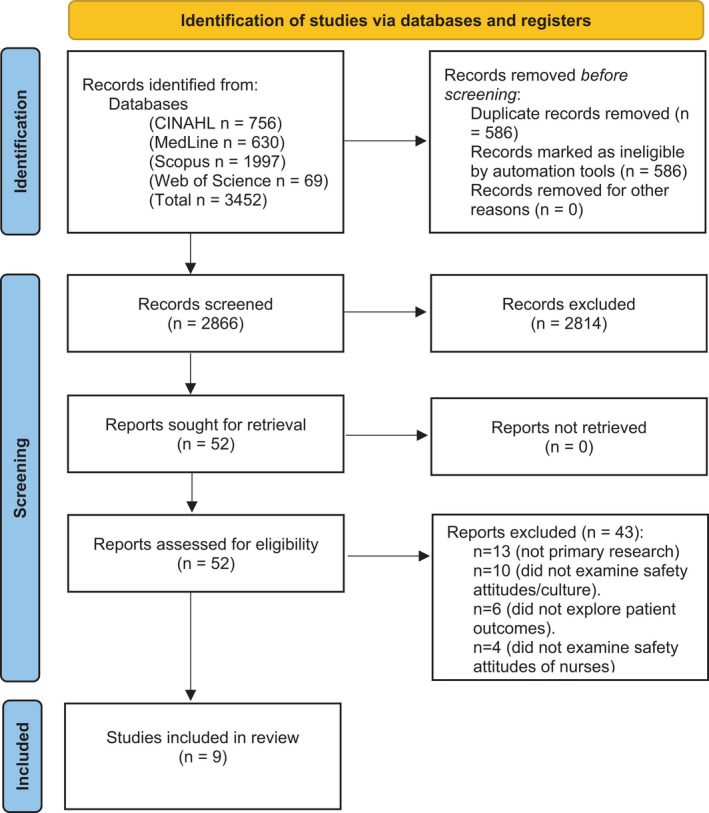
PRISMA Flow Diagram (Page et al., 2021)

The search identified 3,452 studies. EndNote was used for bibliographic management (The EndNote Team, 2013). The screening process was conducted in sequential steps (see PRISMA flow diagram, Figure 1; Page et al., 2021). After removal of duplicates, a total of 2,866 studies underwent title and abstract screening by one author (FKA) and 2,814 were not relevant and were excluded. The remaining fifty‐two full‐text studies were then independently reviewed by all authors and nine were deemed eligible for inclusion. No disagreements arose over inclusion and exclusion criteria, and the nine studies were retained for quality appraisal.

### 2.6 Quality appraisal

The quality of included studies was assessed using the JBI critical appraisal tool for analytical cross‐sectional studies. The tool includes eight questions with response options of Yes, No, Unclear or Not applicable (Moola et al., 2020). All appraisal scores were converted to a percentage, and scores of yes for ≥70% of questions were considered low risk of bias; 50%–69% moderate risk of bias; and ≤49% high risk of bias (Melo et al., 2018). All three authors critically appraised each study independently and then met to compare their evaluations. There were no disagreements, and all nine studies were included in the review. Quality appraisal results are documented in Table 2, and the checklist is provided in Appendix [Link], [Link].

**TABLE 2:** Summary table

Citation (Year) Country	Design and sample	Risk of bias	Safety culture measures	Patient outcomes measures	Key findings relating to safety culture
Ausserhofer et al. (2013) Switzerland	Cross‐sectional study in 35 Swiss acute‐care hospitals Registered nurses (RNs) (*n* = 1,630) Patients (*n* = 997)	Low	Safety Organizing Scale (9 items).	Nurse perceptions of adverse events: Patient falls. Medication errors. Pressure injury. Urinary tract infection. Bloodstream infection. Pneumonia. Patient satisfaction with care.	‐ Mean level of unit safety culture was 5.11 (0.49), 7‐point scale (higher scores indicate better safety culture). ‐ There was no significant relationship between safety culture and any patient outcomes.
Brown and Wolosin (2013) United States of America	Cross‐sectional study in 9 hospitals Nursing units (*n* = 37)	Low	Hospital Survey on Patient Safety Culture (42 items).	Incident/risk management data: Patient falls. Falls with injury (Moderate Injury or above). Pressure injury stage2+.	‐ Mean level of unit safety culture was 3.94 (0.44), 5‐point scale (higher scores indicate better safety culture). ‐ Stronger teamwork within units led to fewer reported falls r = –0.32, *p* <.05. ‐ Stronger overall safety culture led to fewer pressure injuries r = –0.34, *p* <.05. ‐ More management support led to higher numbers of reported falls r = +0.35, *p* <.03.
Han et al. (2020) South Korea	Cross‐sectional study in 2 hospitals Nurses (*n* = 212)	Low	Hospital Survey on Patient Safety Culture (42 items).	Nurse perceptions of adverse events: Patient falls. Medication errors. Pressure injuries. Use of physical restraints for ≥8 hr. Surgical site infection. Urinary tract infection. Central line‐associated bloodstream infections. Ventilator‐associated pneumonia	‐ Mean level of individual safety attitude was 3.49 (0.58), 5‐point scale (higher scores indicate better safety culture). ‐ Negative relationship between stronger safety culture subscale scores and fewer patient outcomes (except central line‐associated bloodstream infections).
Hessels et al. (2019) United States of America	Cross‐sectional study in 5 hospitals Nursing units (*n* = 29) Registered nurses (*n* = 311)	Low	Hospital Survey on Patient Safety Culture (44 items).	Incident/risk management data: Patient falls. Medication Variance. Quality‐of‐care concern. Vascular access device (VAD) events.	‐ Mean level of unit safety culture was 3.52 (0 0.30), 5‐point scale (higher scores indicate better safety culture). ‐ The unit safety culture subscale of management support for patient safety was a predictor of VAD events (R^2^ = 15%, *p* =.01)
Hofmann and Mark (2006) United States of America	Cross‐sectional study in 42 hospitals Nursing units (*n* = 81) Registered nurses (*n* = 1,127)	Low	Zohar Safety Climate Scale (9 items) and Error Orientation Scale (13 items).).	Incident/risk management data: Medication errors. Urinary tract infections. Patient satisfaction with care.	‐ Mean level of unit safety culture was 3.62 (0.26), 5‐point scale (higher scores indicate better safety culture). ‐ Negative relationship between stronger overall safety culture and fewer medication errors (r = −1.51, *p* <.05) and urinary tract infections (r = −1.27, *p* <.05). ‐ Positive relationship between stronger overall safety culture and higher patient satisfaction (r = 0.27, *p* <.01).
Lee et al. (2018) Canada	Cross‐sectional study in 63 hospitals Registered nurses (*n* = 1,053)	Low	Hospital Survey on Patient Safety Culture (7 items).	Nurse perceptions of adverse events: Patient falls with injury. Medication errors. Urinary tract infections . Quality of care.	‐ Negative relationship between stronger hospital safety culture and fewer medication errors (OR =0.36, CI =0.16–0.80), patient falls with injury (OR =0.42, CI =0.18–0.96), and UTIs (OR =0.40, CI =0.18–0.86).
Olds et al. (2017) United States of America	Cross‐sectional study in 600 hospitals Registered nurses (*n* = 27,009) Patients (*n* = 852,974)	Low	Hospital Survey on Patient Safety Culture (7 items).	Coded medical record data In‐hospital mortality.	‐ Mean level of hospital safety culture was 55.0%, (8.7%) (higher percentages indicate better safety culture). ‐ Negative relationship between stronger hospital safety culture (in individual model) and lower mortality (OR =0.92, CI=0.89–0.95, *p* <.001).
Taylor et al. (2012) United States of America	Cross‐sectional study in 1 hospital Nursing units (*n* = 29) Registered nurses (*n* = 723) Patients (*n* = 28,876)	Low	Safety Attitude Questionnaire (27 items).	Incident/risk management data: Patient falls. Coded medical record data. Pressure injuries. Embolism/ deep vein thrombosis (PE/DVT).	‐ Negative relationship between stronger unit subscales of “safety climate” and fewer pressure injuries (OR=0.52, CI =0.29–0.92, *p* <.05). ‐ Negative relationship between stronger unit subscales of “teamwork climate” and fewer pressure injuries (OR=0.56, CI 0.38–0.82, *p* <.01).
Wang et al. (2014) China	Cross‐sectional study in 7 hospitals Nursing units (*n* = 28) Registered nurses (*n* = 463)	Low	Hospital Survey on Patient Safety Culture (42 items).	Nurse perceptions of adverse events: Patient falls. Medication error. Pressure injuries. Physical restraints (> 8 hr). Surgical wound infection. Infusion or transfusion reaction. Patients or family complaints.	‐ Mean level of individual safety attitude was 3.46 (0.60), 5‐point scale (higher scores indicate better safety culture). ‐ Negative relationship between stronger safety culture subscales and fewer patient outcomes (except patient falls).

### 2.7 Data abstraction

Data were extracted from each study into a summary table (Table 2) by two authors (FKA and JS) independently. This included details on the citation (year) country, design and sample, risk of bias, safety culture measures, patient outcomes measures and key findings relating to safety attitudes and culture.

### 2.8 Data analysis and synthesis

A meta‐analysis was not feasible due to heterogeneity in how patient outcomes were defined and examined and variation in methodological approaches used to collect both safety attitudes and patient outcomes data. Therefore, data were synthesized narratively using the relevant items from the Synthesis Without Meta‐analysis (SWiM) guidelines (Campbell et al., 2020). Data related to nurses' safety attitudes were compared and summarized using six domains. Patient outcomes were grouped and narratively synthesized.

## 3 RESULTS

### 3.1 Characteristics of included studies

Of the nine included studies, five were undertaken in the United States of America (USA; *N* = 5), with one each from Canada, Switzerland, South Korea and China. All studies used cross‐sectional designs. Data were reported at the unit level in five studies (Ausserhofer et al., 2013; Brown & Wolosin, 2013; Hessels et al., 2019; Hofmann & Mark, 2006; Taylor et al., 2012), at hospital level in two studies (Lee et al., 2018; Olds et al., 2017) and at individual nurse level in two studies (Han et al., 2020; Wang et al., 2014).

### 3.2 Safety attitudes of nurses

All included studies rated safety attitudes as positive. The highest overall mean score for safety attitudes was 3.94 (out of 5), *SD*= 0.44 (Brown & Wolosin, 2013) using the 42‐item version of the Hospital Survey on Patient Safety Culture (HSOPSC). The lowest mean score was 55.0%, *SD* = 8.7%, using the seven‐item version of the HSOPSC (Olds et al., 2017). A positive score on teamwork elements was identified in six studies (Ausserhofer et al., 2013; Brown & Wolosin, 2013; Han et al., 2020; Hessels et al., 2019; Taylor et al., 2012; Wang et al., 2014). One study reported that teamwork and collaboration between staff were sub‐optimal (Olds et al., 2017). Stress recognition was examined in three studies and all reported positive scores (Han et al., 2020; Hessels et al., 2019; Wang et al., 2014). Most studies reported that management provides appropriate responses when errors are reported (Ausserhofer et al., 2013; Brown & Wolosin, 2013; Han et al., 2020; Hessels et al., 2019; Hofmann & Mark, 2006; Taylor et al., 2012; Wang et al., 2014). However, one study identified that although patient safety was perceived to be a top priority of management, nurses were scared of making mistakes and feared punishment from management which may lead to some errors not being reported (Olds et al., 2017). The importance of hospital working conditions and the presence of a learning culture was positively reported in all studies. Morale was explored in only one study and staff reported positive morale which contributed to positive safety attitudes (Taylor et al., 2012).

The HSOPSC was the most commonly used instrument to assess nurses' safety attitudes (*N* = 6; Brown & Wolosin, 2013; Han et al., 2020; Hessels et al., 2019; Lee et al., 2019; Olds et al., 2017; Wang et al., 2014). The original HSOPSC was developed by the Agency for Healthcare Research and Quality (Sorra et al., 2016), and three different versions were used in the six included studies. The 42‐item version was used in three studies (Brown & Wolosin, 2013; Han et al., 2020; Wang et al., 2014), one study used 44 items (Hessels et al., 2019) and two studies used seven items (Lee et al., 2018; Olds et al., 2017). Four other instruments, namely the Safety Organizing Scale (SOS), Safety Attitudes Questionnaire (SAQ), Zohar Safety Climate Scale (ZSCS) and Error Orientation Scale (EOS), were also used to measure nurses' safety attitudes. The SOS includes nine items that assess nurses' engagement in safety behaviours at the unit level (Ausserhofer et al., 2013). The SAQ used by Taylor et al. (2012) included 36 items and five subscales. One study used both the ZSCS (9 items) and the EOS (13 items) to measure nurses' perceptions about safety culture (Hofmann & Mark, 2006). All instruments measured safety attitudes using five‐ or seven‐point Likert scales. Results were presented as either an overall mean score, composite scores for each domain or converted to a percentage. Higher scores indicated a stronger safety culture or safety attitude in all studies. The characteristics of the instruments used to assess nurses' safety attitudes are presented in Table 3.

**TABLE 3:** Characteristics of the instruments used to assess nurses' safety attitudes in the included studies

Instrument	Studies using this instrument	Constructs / Domains being examined	Scoring	Reliability
Hospital Survey on Patient Safety Culture (HSOPSC) (7 items)	Lee et al. (2018) Olds et al. (2017)	7 items of HSOPSC; ‐ Staff feel like their mistakes are held against them. ‐ Important patient care information is often lost during shift changes. ‐ Things “fall between the cracks” when transferring patients from one unit to another. ‐ Staff feel free to question the decisions or actions of those in authority. ‐ In this unit, we discuss ways to prevent errors from happening again. ‐ We are given feedback about changes put into place based on event reports. ‐ The actions of hospital/organization management show that patient safety is a top priority.	5‐point Likert scale Agreement ranges from 1 = strongly disagree to 5 = strongly agree. or Frequency ranges from 1 = never to 5 = always. Mean score above 2.5 (or 50% for percentages) indicates a positive safety culture	Cronbach α was between 0.76 to 0.80
Hospital Survey on Patient Safety Culture (HSOPSC)	Brown and Wolosin (2013) Han et al. (2020) Hessels et al. (2019 Wang et al. (2014)	12 subscales of HSOPSC measured by 42 and 44 items. ‐ Teamwork within units. ‐ Supervisor/manager expectations and actions promoting patient safety. ‐ Organizational learning continuous improvement. ‐ Management support for patient safety. ‐ Overall perceptions of patient safety. ‐ Feedback and communication about error. ‐ Communication openness. ‐ Frequency of events reported. ‐ Teamwork across units. ‐ Staffing. ‐ Handoffs and transition. ‐ Non‐punitive response to error.	5‐point Likert scale Agreement ranges from 1 = strongly disagree to 5 = strongly agree. or Frequency ranges from 1 = never to 5 = always. Mean score above 2.5 indicates a positive safety culture	Cronbach α was between 0.64 to 0.95
Safety Organizing Scale (SOS)	Ausserhofer et al. (2013)	Unidimensional instrument, consisting of nine items ‐ We have a good “map” of each other's talents and skills. ‐ We talk about mistakes and ways to learn from them. ‐ We discuss our unique skills with each other so we know who on the unit has relevant specialized skills and knowledge. ‐ We discuss alternatives as to how to go about our normal work activities. ‐ When giving report to an oncoming nurse, we usually discuss what to look out for. ‐ When attempting to resolve a problem, we take advantage of the unique skills of our colleagues. ‐ We spend time identifying activities we do not want to go wrong. ‐ When errors happen, we discuss how we could have prevented them. ‐ When a patient crisis occurs, we rapidly pool our collective expertise to attempt to resolve it.	7‐points Likert scale Likert scale range from 1 = not at all, 2 = to a very limited extent, 3 = to a limited extent 4 = to a moderate extent 5 = to a considerable extent 6 = to a great extent 7 = to a very great extent Mean score above 3.5 indicates a positive safety culture.	Cronbach's alpha reported for previous studies was >0.79
Safety Attitudes Questionnaire (SAQ)	Taylor et al. (2012)	Five subscales of SAQ measured by 27 items. ‐ Teamwork Climate. ‐ Safety Climate. ‐ Morale. ‐ Perception of Management. ‐ Working Conditions.	5‐point Likert scale Range from 1 = strongly disagree to 5 = strongly agree. Scores converted to a percentage scale using the following method: Responses are converted to 1 = 0%, 2 = 25%, 3 = 50%, 4 = 75% and 5 = 100% Mean percentage above 50% indicates a positive safety attitude.	Cronbach's alpha for SAQ domain ‐ Teamwork Climate =0.77 ‐ Safety Climate =0.75 ‐ Morale =0.87 ‐ Perception of Management =0.75 ‐ Working Conditions =0.69
Zohar Safety Climate Scale (ZSCS) (9 items).	Hofmann and Mark (2006)	Three subscales of ZSCS measured by 9 items. ‐ Job duties (three items). ‐ Social standing (three items). ‐ Management attitudes (three items).	5‐point Likert scale Range from 1 = strongly disagree to 5 = strongly agree. Mean score above 2.5 indicates a positive safety culture.	Internal consistency reliability ‐ Job duties =0.97 ‐ Social standing =0.71 ‐ Management attitude =0.79
Error Orientation Scale (EOS) (13 items)	Hofmann and Mark (2006)	Three subscales of EOS measured by 13 items. ‐ Reveal errors (four items). ‐ Communication about errors (four items). ‐ Think about errors (five items).	5‐point Likert scale Range from 1 = strongly disagree to 5 = strongly agree. Mean score above 2.5 indicates a positive safety culture.	Internal consistency reliability ‐ Reveal errors =0.83 ‐ Communication about errors =0.86 ‐ Thinking about errors =0.92.

### 3.3 Patient outcomes data

The included studies examined patient falls (*N* = 7), medication errors (*N* = 6), pressure injuries (*N* = 5), healthcare‐associated infections (*N* = 5), patient mortality (*N* = 1), patient satisfaction (*N* = 3), physical restraint (*N* = 2) and vascular access device complications (*N* = 2). There were four primary sources of patient outcomes data reported in the included studies: (a) coded medical record data (also referred to as discharge datasets) (*N* = 2), (b) incident management systems (*N* = 3), (c) nurse perceptions of adverse events (*N* = 4) and (d) patient perceptions of safety (*N* = 2).

#### 3.3.1 Patient falls

Seven studies examined patient falls and three reported significant associations between nurses' positive safety attitudes and a reduction in patient falls (Brown & Wolosin, 2013; Han et al., 2020; Lee et al., 2018). Brown and Wolosin (2013) identified that nursing units with positive teamwork had lower numbers of patient falls recorded at the unit level (r = −0.32, *p* < .05). In addition, higher levels of management support for patient safety led to nurses accurately reporting the patient falls that occurred (r = +0.35, *p* < .03) (Brown & Wolosin, 2013). This indicates that when nurse managers create supportive cultures, individual nurses are willing to report patient falls which facilitates development of a learning culture. Han et al. (2020) stated that nurses recorded fewer patient falls if the unit had high scores in the subscales of “teamwork within units” (OR = 0.23, CI = 0.07–0.76), “teamwork across units” (OR = 0.29, CI = 0.09–0.93), “communication openness” (OR = 0.25, CI = 0.08–0.93) and “supervisor/manager expectations and actions promoting patient safety” (OR = 0.33, CI = 0.12–0.94). Additionally, when nurses experienced a strong safety culture, there was a 58% drop in reporting of patient falls (OR = 0.42, CI = 0.18–0.96; Lee et al., 2018). However, there were no significant association between nurses' positive safety attitudes and a reduction in patient falls in four studies (Ausserhofer et al., 2013; Hessels et al., 2019; Taylor et al., 2012; Wang et al., 2014).

#### 3.3.2 Medication errors

Medication errors were examined in six studies. Four of the included studies showed significant association between nurses' positive safety attitudes and reduced medication errors (Han et al., 2020; Hofmann & Mark, 2006; Lee et al., 2018; Wang et al., 2014). Two studies used incident management data to measure medication errors (Hessels et al., 2019; Hofmann & Mark, 2006) and one of these identified that nursing units with positive safety culture recorded fewer medication errors (r = −1.51, *p* < .05; Hofmann & Mark, 2006). In contrast, individual nurses reported that medication errors occurred less frequently when the manager demonstrated actions that promoted patient safety (OR = 0.36, CI = 0.13–0.96; Han et al., 2020). Similarly, nurses in settings with a strong safety culture were 64% less likely to record administering the wrong medication (time and dose) (OR = 0.36, CI = 0.16–0.80; Lee et al., 2018). Less frequent medication errors were also reported by individual nurses who indicated that they routinely reported all events (OR = 0.69, *p* = .021) and who believed that patient safety was supported by management (OR = 0.51, *p* = .006; Wang et al., 2014).

#### 3.3.3 Pressure injuries

Of the five studies that examined pressure injuries, four studies reported significant association between nurses' positive safety attitudes and reduced numbers of pressure injuries (Brown & Wolosin, 2013; Han et al., 2020; Taylor et al., 2012; Wang et al., 2014). Nursing units with positive safety culture reported fewer pressure injuries (r = −0.34, *p* < .05; Brown & Wolosin, 2013). Likewise, pressure injuries were less likely to be documented in the medical records when the nursing unit had a positive safety culture (OR = 0.52, CI = 0.29–0.92); and positive teamwork (OR = 0.56, CI = 0.38–0.82; Taylor et al., 2012). Higher ratings of management support for patient safety (OR = 0.37, CI = 0.16–0.88) and communication openness (OR = 0.40, CI = 0.16–0.97) led to individual nurses reporting fewer pressure injuries (Han et al., 2020). Similarly, the number of pressure injuries reported by nurses reduced with higher mean scores for organizational learning (OR = 0.24, *p* = .002), communication about error (OR = 0.41, *p* = .037), non‐punitive response to error (OR = 0.66, *p* = .045) and tendency of individual nurses to report all errors/events (OR = 0.63, *p* = .006; Wang et al., 2014).

#### 3.3.4 Healthcare‐associated infections

Healthcare‐associated infections were examined in five studies. Four studies reported significant association between nurses' positive safety attitudes and decreased occurrence of healthcare‐associated infections (Han et al., 2020; Hofmann & Mark, 2006; Lee et al., 2018; Wang et al., 2014). Analysis of data reported in incident management reports indicated that nursing units with a positive safety culture recorded lower incidence of urinary tract infections (r = −1.27, *p* < .05) (Hofmann & Mark, 2006). Nurses working in hospitals with a positive safety culture were 60% less likely to report frequent urinary tract infections (OR =0.40, CI = 0.18–0.86; Lee et al., 2018). Han et al. (2020) reported that surgical site infections reduced when nursing units had higher mean scores for teamwork (OR = 0.51, CI = 0.27–0.97), communication about error (OR = 0.52, CI = 0.27–0.97) and communication openness (OR = 0.51, CI = 0.28–0.93). Wang et al. (2014) found that a higher mean score for effectiveness of hospital handoffs and transitions was associated with fewer surgical wound infections (OR = 0.477, *p* = .004). Nurses' perceptions of the frequency of urinary tract infections reduced with higher mean scores for actions promoting patient safety by managers (OR = 0.35, CI = 0.18–0.68), communication about error (OR = 0.43, CI = 0.22–0.83), communication openness (OR = 0.41, CI = 0.21–0.77) and teamwork across units (OR = 0.46, CI = 0.23–0.93; Han et al., 2020). Ventilator‐associated pneumonia reduced when nurses felt that patient safety was supported by management (OR = 0.55, CI = 0.31–0.97), there was teamwork across units (OR = 0.47, CI = 0.24–0.93) and a non‐punitive response to error (OR =2.08, CI =1.04–4.16) (Han et al., 2020).

#### 3.3.5 Mortality

Only one study examined nurses' safety culture and patient mortality. Olds et al. (2017) identified that positive safety culture was associated with a decreased risk of death (OR = 0.92, CI = 0.89–0.95).

#### 3.3.6 Patient satisfaction

Four studies examined patient satisfaction with positive results between nurses' safety attitude and patient satisfaction reported in three studies (Hessels et al., 2019; Hofmann & Mark, 2006; Wang et al., 2014). Nursing units with positive safety culture reported higher patient satisfaction (r = 0.27, *p* < .01) (Hofmann & Mark, 2006). Nurse perception of the frequency of patient complaints was lower in hospitals that provided more continuous learning (OR = 0.36, *p* = .01) and actions that improve and enhance patient safety (OR = 0.64, *p* = .02; Wang et al., 2014). Hessels et al. (2019) reported strong correlations between quality‐of‐care issues, such as delays in responding to patient conditions, communication issues, inappropriate treatment and handoff issues, and the HSOPSC sub‐scales of non‐punitive response to error (R^2^ = 26%, *p* =.02), tendency of individual nurses to report all errors/events (R^2^ = 21%, *p* = .04), and feedback and communication about error (R^2^ = 4%, *p* = .03).

#### 3.3.7 Physical restraints

The use of physical restraints was examined in two studies (Han et al., 2020; Wang et al., 2014) using nurse perceptions of adverse events. Nurse reports of physical restraints being used for more than eight hours reduced when nurses worked in hospitals that had optimal organizational learning (OR = 0.40, *p* = .019) and effective communication (OR = 0.54, *p* = .010; Wang et al., 2014). Similarly, restraints use for more than eight hours decreased when patient safety was promoted by managers (OR = 0.39, CI = 0.19–0.79) and when there was increased collaboration between staff during patient handovers (OR = 2.02, CI = 1.02–3.98; Han et al., 2020).

#### 3.3.8 Vascular access device

The correlation between vascular access device complications and nurses' safety attitudes was examined in two studies (Hessels et al., 2019; Wang et al., 2014). Wang et al. (2014) found that a higher mean score for handovers and transitions in care (OR = 0.52, = 0.34), communication about error (OR = 0.73, *p* = .041) and management support for patient safety (OR = 0.50, *p* = .027) were significantly related to a reduction in the frequency of nurses' reporting infusion events and transfusion reactions. In addition, management support for patient safety was a positive predictor of lower numbers of vascular access device events being reported within the incident management system (R^2^ = 15%, *p* = .01; Hessels et al., 2019).

## 4 DISCUSSION

The findings from this systematic review indicate that nurses' positive safety attitudes lead to improved outcomes for patients in acute‐care hospital settings. Despite purposely searching for studies that examined patient outcomes, most included studies examined adverse events in isolation from other outcome measures. This focus on adverse events among included studies is also evident in the literature on nursing‐sensitive patient outcomes (Sim et al., 2018) and may relate to the availability of data and the presence of valid metrics to quantify these events. Only four of the nine included studies explored patient satisfaction (Ausserhofer et al., 2013; Hofmann & Mark, 2006; Wang et al., 2014) and quality‐of‐care issues (Hessels et al., 2019) within this review. Other indicators such as length of stay, failure to rescue and readmissions may also be influenced by nurses' safety attitudes; however, the absence of data on these topics was surprising.

Nurses play a key role within healthcare teams, and the safety attitudes of nurses have an impact on the safety culture of the unit and hospital. This review identified nine studies that examined the impact of nurses' safety attitudes on patient outcomes in acute‐care hospital settings. Only a comparatively small number of observational studies met the inclusion criteria on what is arguably an important and emerging area of interest. This provides a strong justification for the need for further research to investigate the impact of nurses' safety attitudes on patient outcomes in the acute‐care setting. Nonetheless, the included studies provide important evidence about the influence of nurses' safety attitudes on patient outcomes.

This review identified strong associations between nurses' positive safety attitudes and a reduction in the key patient outcomes of falls, medication errors, pressure injuries and healthcare‐associated infections. This is an important finding since these four patient outcomes are widely accepted to be reflective of the quality of care provided in acute‐care settings (Australian Commission on Safety & Quality in Health care, 2017). For example, fall‐related injuries, medication errors and infections are the most commonly reported adverse events in acute‐care settings in Australia (Australian Institute of Health & Welfare, 2018). Similarly, pressure injuries are considered a preventable adverse event by national and international groups such as the Agency for Healthcare Research and Quality (Alshahrani et al., 2021; Chou et al., 2013), Australian Commission on Safety and Quality in Healthcare (Australian Commission on Safety & Quality in Health Care, 2019) and the US Joint Commission on Accreditation in Healthcare Organizations (The Joint Commission, 2016). Consequently, financial penalties are increasingly being imposed on healthcare providers to acknowledge the gravity and preventability of pressure injuries (Nguyen et al., 2015). While a number of initiatives such as environmental modifications (Clay et al., 2018), staff education, risk assessments (Al‐Otaibi et al., 2019) and electronic medication management systems (The Clinical Knowledge Network, 2017) have been implemented in an effort to reduce patient harm, the results have been mixed. Safety attitudes may therefore be an important variable and worthy of further study.

Nurses' positive safety attitudes and reduction in mortality, use of physical restraints, vascular access device complications and higher patient satisfaction were also examined within the included studies. The findings on these patient outcomes are limited in that they were based on a small number of studies; however, some of the associations are consistent with previous literature on the nursing practice environment. A systematic review and meta‐analysis reported that positive nursing work environment was associated with a lower probability of mortality and higher patient satisfaction (Lake et al., 2019). Another systematic review found an association between strong working conditions and a reduction in the rate of mortality; however, there was no association with patient satisfaction (Bae, 2011). Considering a few studies have examined the impact of nurses' safety attitudes on these patient outcomes, these outcomes require further investigation.

The findings from this systematic review are based on studies that were observational in nature, with many studies relying on self‐reported data. In addition, nurses' safety attitudes were assessed using a variety of different tools and at different organizational levels. Five validated instruments were used in the nine included studies. These differences in how nurses' safety attitudes were measured led to inconsistencies, which limit our understanding of the association between nurses' safety attitudes and patient outcomes. Furthermore, analysis at different organizational levels (individual nurse, nursing unit/ward and hospital) further limits our understanding of these associations. Two studies that analysed safety attitudes at the individual level, using the full version of HSOPSC and nurses' perception of adverse events, found statistically significant relationships (Han et al., 2020; Wang et al., 2014). Hospital‐level analysis was used in one study using the seven‐item version of HSOPSC and nurses' perception of adverse events (Lee et al., 2018) and another using administrative data (Olds et al., 2017) and both studies identified statistically significant relationships. In contrast, studies that used unit‐level analysis reported limited associations between nurses' safety attitudes and patient outcomes. For example, patient falls were not significantly associated with the nursing unit safety culture in three studies (Ausserhofer et al., 2013; Hessels et al., 2019; Taylor et al., 2012). This contrasts with studies that evaluated patient falls at the individual (Han et al., 2020) and hospital level (Lee et al., 2018). Interestingly, individual safety attitudes and nurses' perceptions of adverse event data remained consistent when the data were aggregated to the hospital level.

## 5 LIMITATIONS

Although a comprehensive search strategy was used, it is possible that not all relevant studies were included in this review. More than half of the studies were conducted in the USA; therefore, further international research is required. Wide variation in the number of instruments used, and the source of patient outcomes data was noted. For instance, data related to the frequency of patient falls was collected using a cross‐sectional survey in some studies (Ausserhofer et al., 2013; Han et al., 2020; Lee et al., 2018; Wang et al., 2014) and via administrative datasets in others (Brown & Wolosin, 2013; Hessels et al., 2019; Taylor et al., 2012). This heterogeneity impacts upon our understanding of the complex relationship between nurses' safety attitudes and patient outcomes. In addition, nurses' perceptions of adverse events may also be influenced by how the individual nurse's perceptions of outcomes were measured and analysed. Two included studies (Han et al., 2020; Wang et al., 2014) dichotomized participant responses into groups of “never happened” or “happened” (thereby comparing “never happened” with the grouping of the other six categories including “happened several times a year” to “happened daily”) during data analysis. Other studies dichotomized the frequency of events so that events that never occurred or rarely occurred (once per month or a few times per year) and those that occurred occasionally or frequently were grouped together (a few times per month, once a week, a few times a week and every day) (Aiken et al., 2017; Lake et al., 2016). These different approaches led to variability in results between studies and made the synthesis of findings challenging. Furthermore, all of the included studies were cross‐sectional in design, which makes it difficult to make causal inference between nurses' safety attitudes and patient outcomes.

## 6 CONCLUSION

Evidence from the included articles shows that acute‐care hospitals and units with positive safety culture report fewer adverse patient outcomes. These findings suggest that nurses' positive safety attitudes have the potential to improve patient outcomes and prevention of harm to patients in acute‐care hospital settings. Individual nurses must be empowered to develop and maintain positive safety attitudes as these attitudes contribute to the safety culture of the ward or unit where they work. The collective safety culture of a ward is influenced by the nurse managers approach to safety. However, future research to fully explore the relationship between nurses' safety attitudes and patient outcomes using stronger research designs is warranted.

## 7 RELEVANCE TO CLINICAL PRACTICE

This systematic review highlights that nurses with positive safety attitudes substantially improve patient safety in acute‐care settings. Teamwork, collaboration and communication among nurses in the area of professional practice lead to a reduction in the occurrence of adverse patient outcomes. Nurse managers can improve patient outcomes by focusing on improving nurses' safety attitudes. Nurse managers play essential roles in building and promoting nurses' safety attitudes through having a non‐punitive response to error, creation of trusting relationships and developing environments where individuals can learn from error.

## CONFLICT OF INTEREST

No conflict of interest has been declared by the authors.

## AUTHOR CONTRIBUTIONS

FKA involved in searching databases, collecting data and writing of the initial draft. FKA, JS and SL independently screened and apprised the full text included studies. JS and SL comprehensively revised the content of review after each draft. All authors approved the final version to be submitted for publication.

## ETHICAL APPROVAL

This was a systematic review of previously published research, and thus, Research Ethics Committee approval was not required.

## Supporting information

Supplementary Material

Supplementary Material

## Data Availability

The authors confirm that the data supporting the findings of this study are available within the article [and/or] its [Link], [Link].
