# Supplementary material for: Systematic review: Nurses' safety attitudes and their impact on patient outcomes in acute‐care hospitals
Source: Nurs Open. 2021 Sep 19;9(1):30–43. doi: 10.1002/nop2.1063 (PMC8685891; doi:10.1002/nop2.1063)
Supplement: Supplementary file 1 — Supplementary Material [file NOP2-9-30-s001.docx]

Supplemental File A: Quality Appraisal Scores

| Citation | Criterion - JBI Critical Appraisal Checklist for Analytical Cross Sectional Studies | | | | | | | | Results (%) |
| --- | --- | --- | --- | --- | --- | --- | --- | --- | --- |
|  | 1 | 2 | 3 | 4 | 5 | 6 | 7 | 8 |  |
| Ausserhofer et al. (2013) | Y | Y | Y | Y | Y | Y | Y | Y | 8/8  (100%) |
| Brown and Wolosin (2013) | Y | Y | Y | Y | U | U | Y | Y | 6/8  (75%) |
| Han et al. (2020) | Y | Y | Y | Y | Y | Y | Y | Y | 8/8  (100%) |
| Hessels et al. (2018) | Y | Y | Y | Y | Y | U | Y | Y | 7/8  (87.5%) |
| Hofmann and Mark (2006) | Y | Y | Y | Y | U | U | Y | Y | 6/8  (75%) |
| Lee et al. (2018) | Y | Y | Y | Y | Y | Y | Y | Y | 8/8  (100%) |
| Olds et al. (2017) | Y | Y | Y | Y | Y | U | Y | Y | 7/8  (87.5%) |
| Taylor et al. (2012) | Y | Y | Y | Y | Y | U | Y | Y | 7/8  (87.5%) |
| Wang et al. (2014) | Y | Y | Y | Y | Y | Y | Y | Y | 8/8  (100%) |
| Y = yes; N = no; U = unclear  1. Were the criteria for inclusion in the sample clearly defined?  2. Were the study subjects and the setting described in detail?  3. Was the exposure measured in a valid and reliable way?  4. Were objective, standard criteria used for measurement of the condition?  5. Were confounding factors identified?  6. Were strategies to deal with confounding factors stated?  7. Were the outcomes measured in a valid and reliable way?  8. Was appropriate statistical analysis used? | | | | | | | | | |
